# Transcriptional analysis of highly syntenic regions between *Medicago truncatula *and *Glycine max *using tiling microarrays

**DOI:** 10.1186/gb-2008-9-3-r57

**Published:** 2008-03-19

**Authors:** Lei Li, Hang He, Juan Zhang, Xiangfeng Wang, Sulan Bai, Viktor Stolc, Waraporn Tongprasit, Nevin D Young, Oliver Yu, Xing-Wang Deng

**Affiliations:** 1 Department of Molecular, Cellular, and Developmental Biology, Yale University, New Haven, CT 06520, USA; 2 National Institute of Biological Sciences, Beijing 102206, China; 3 Peking-Yale Joint Research Center of Plant Molecular Genetics and Agrobiotechnology, Peking University, Beijing 100871, China; 4 Donald Danforth Plant Science Center, St Louis, MO 63132, USA; 5 College of Life Sciences, Capital Normal University, Beijing 100037, China; 6 Genome Research Facility, NASA Ames Research Center, Moffett Field, CA 94035, USA; 7 Department of Plant Pathology, University of Minnesota, St Paul, MN 55108, USA; 8 Current address: Department of Biology, University of Virginia, Charlottesville, VA 22904, USA

## Abstract

**Background:**

Legumes are the third largest family of flowering plants and are unique among crop species in their ability to fix atmospheric nitrogen. As a result of recent genome sequencing efforts, legumes are now one of a few plant families with extensive genomic and transcriptomic data available in multiple species. The unprecedented complexity and impending completeness of these data create opportunities for new approaches to discovery.

**Results:**

We report here a transcriptional analysis in six different organ types of syntenic regions totaling approximately 1 Mb between the legume plants barrel medic (*Medicago truncatula*) and soybean (*Glycine max*) using oligonucleotide tiling microarrays. This analysis detected transcription of over 80% of the predicted genes in both species. We also identified 499 and 660 transcriptionally active regions from barrel medic and soybean, respectively, over half of which locate outside of the predicted exons. We used the tiling array data to detect differential gene expression in the six examined organ types and found several genes that are preferentially expressed in the nodule. Further investigation revealed that some collinear genes exhibit different expression patterns between the two species.

**Conclusion:**

These results demonstrate the utility of genome tiling microarrays in generating transcriptomic data to complement computational annotation of the newly available legume genome sequences. The tiling microarray data was further used to quantify gene expression levels in multiple organ types of two related legume species. Further development of this method should provide a new approach to comparative genomics aimed at elucidating genome organization and transcriptional regulation.

## Abstract 2

The comparative transcriptional analysis of highly syntenic regions in six different organ types between *Medicago truncatula* (barrel medic) and *Glycine max* (soybean), using nucleotide tiling microarrays, provides insights into genome organization and transcriptional regulation in these legume plants.

## Background

The rapidly increasing number of genome and transcript sequences in recent years is having two marked, complementary effects on the relatively new discipline of plant genomics and transcriptomics. The newly available sequences need to be fully annotated to identify all the functional and structural elements. Because genome annotation is a reiterative process that is heavily dependent on large-scale, high-throughput experimental data, each additional genome sequence comes as a new challenge. On the other hand, the availability of multiple genomic and transcriptomic datasets fosters comparative analyses that improve structural annotation of the genomes and generate new insight into the function and evolution of protein-coding and non-coding regions of the genomes.

One approach to systematically characterize genome transcription is to use high feature-density tiling microarrays on which a given genome sequence is represented [1,2]. Genome tiling arrays have been used in a number of model species for which the full genome sequence is available [3-8]. Results from these studies have shown that for well-documented transcripts, such as those of polyadenylated RNAs from annotated genes, hybridization signals from tiling arrays identify the transcriptional start and stop sites, the locations of introns, and the events of alternative splicing [3-8]. Tiling arrays therefore provide a valuable means for confirming the large number of predicted genes that otherwise lack supportive experimental evidence. However, tiling array signals also reveal a large number of putative novel transcripts for which no conventional explanations are yet available.

With respect to plants, the *Arabidopsis thaliana *genome was the first to be probed by tiling microarrays [5]. Tiling array analysis of the more complex rice genome has been carried out as well [8-10]. The rice tiling array data were used to detect transcription of the majority of the annotated genes. For example, of the 43,914 non-transposable element protein-coding genes from the improved *indica* whole genome shotgun sequence [11], transcription of 35,970 (81.9%) was detected [8]. On the other hand, comprehensive identification of transcriptionally active regions (TARs) from tiling array profiles revealed significant transcriptional activities outside of the annotated exons [8-10]. Subsequent analyses indicate that about 80% of the non-exonic TARs can be assigned to various putatively functional or structural elements of the rice genome, ranging from splice variants, uncharacterized portions of incompletely annotated genes, antisense transcripts, duplicated gene fragments, to potential non-coding RNAs [10].

In addition to detecting transcriptome components, genome tiling arrays in theory can be used to directly quantify the expression levels of individual transcription units. As an alternative approach to the surrogate expression arrays, tiling arrays offer two potential advantages. First, in tiling arrays each transcription unit is interrogated by hundreds of probes according to the actual genomic sequence. This strategy eliminates the need to arbitrarily select a small number of supposedly gene-specific probes and thus alleviates probe bias and improves cross-platform comparability in microarray experiments. Second, measurement of gene expression using tiling arrays allows averaging of the results from multiple probes per gene, which can reduce inconsistent probe behavior and thus provide improved statistical confidence.

Using DNA microarrays to study gene expression in closely related species has become an important approach to identify the genetic basis for phenotypic variation and to trace evolution of gene regulation [12-17]. However, expression levels as well as sequences may differ between species, creating additional technical challenges for inter-species comparisons. Current approaches to control for the effect of sequence divergence are either to mask probes with sequence mismatches [17,18] or to use probes derived from the various species of interest to cancel out the sequence mismatch effect [19,20]. Both approaches, however, rely on a few empirically or computationally selected probes for each gene of interest. Consequently, the effectiveness and accuracy of these approaches is still a matter of debate [18]. In related species for which genome sequences have all been determined, genomic tiling arrays could provide an alternative approach to inter-species comparison of gene expression. Again, the inclusion of multiple probes per transcription unit in tiling arrays could potentially improve the accuracy and fairness of the estimation of gene expression levels in each species, which in turn could improve cross-species comparison of the expression patterns of orthologous genes.

As the third largest family of flowering plants, legumes (Fabaceae) are unique among crop species in their ability to fix atmospheric nitrogen through symbiotic relationships with rhizobia bacteria [21]. Extensive expressed sequence tags have been collected for a number of legume species, including soybean (*Glycine max*), lotus (*Lotus japonicus*), common bean (*Phaseolus vulgaris*), and barrel medic (*Medicago truncatula*) [22,23]. Genomes of barrel medic, soybean, and lotus are being sequenced because all are models for studying nitrogen fixation and symbiosis, tractable to genetic manipulation, and exhibit diploid genetics and modest genome sizes. Both barrel medic and lotus have a diploid genome of approximately 475 Mb while soybean has a diploidized tetraploid genome estimated at 950 Mb [24,25]. Recently, preliminary genome assembly and annotation of barrel medic (Mt2.0) and soybean (Glyma0) became publicly available [26,27]. As a result, legumes are now one of a few plant families in which extensive genome sequences in multiple species are available.

Comparisons of genome sequences have revealed various degrees of synteny (conservation of gene content and order) among species related at different taxonomic levels. For legume plants, early work based on DNA markers demonstrated substantial genome conservation among some Phasoloid species, including mungbean (*Vigna radiata*) and cowpea (*V. unguiculata*) [28], and between *Vigna *and the common bean [29]. Genome-wide gene-based analysis among legumes using a large set of cross-species genetic markers produced chromosome alignments from five species of the Papilionoid subfamily, including barrel medic and soybean [30]. More recently, direct synteny comparison of the finished and anchored genome sequences from barrel medic and lotus was made. Results from this study indicated that three-quarters of the genome of each species may reside in conserved syntenic segments in the genome of the other [25], which share at least ten large-scale synteny blocks that frequently extend the length of whole chromosome arms [26].

Two soybean regions comprising approximately 0.5 Mb each surrounding the soybean cyst nematode resistance loci, *rhg1 *and *Rhg4*, were extensively characterized [31]. Using these sequences, Mudge *et al*. [32] identified the syntenic regions from barrel medic. They found that many predicted genes in the syntenic regions were conserved and collinear between the two species. Here, we used tiling microarray analysis to verify the predicted genes, to identify additional transcripts, and to compare transcription patterns in six different organ types in each species. Our results provide transcriptional support to over 80% of the predicted genes and identified 499 and 660 TARs from barrel medic and soybean, respectively. The gene expression patterns in the six organ types of some collinear genes showed significant differences between the two species despite synteny at the DNA level, demonstrating the usefulness of genomic tiling analysis in comparative genomics.

## Results

### Genes in the syntenic regions between barrel medic and soybean

In a previous study, two regions in the soybean genome comprising approximately 0.5 Mb each surrounding the soybean cyst nematode resistance loci, *rhg1 *and *Rhg4*, were used to identify syntenic regions in the *Medicago *genome [32]. Because there was a 2 cM gap in the first region, these sequences were referred to as synteny blocks 1a, 1b, and 2 [32]. The syntenic regions in barrel medic also totaled about 1 Mb, though they were scattered into smaller contigs. For example, synteny block 1b in barrel medic contained two additional gaps [32]. In barrel medic, there were two segmental duplications (block 2i and 2ii) that were both syntenic to soybean synteny block 2 [32].

Genes were predicted in the 1 Mb barrel medic and soybean sequence contigs using FGENESH [33]. Both the dicot plants (*Arabidopsis*) and the *Medicago *(legume plant) matrixes were used and their outputs compared [33]. Using the legume matrix, 229 and 217 genes were predicted for the barrel medic and soybean sequences, respectively (Additional data file 1). These represent significantly more but shorter genes (exons) compared with the *Arabidopsis *matrix outputs. However, the legume matrix prediction also resulted in more base-pairs in the exons (increases of 10.3% and 8.2% for barrel medic and soybean, respectively; Additional data file 1). These results clearly demonstrate that gene prediction output is sensitive to the training matrix and highlight the importance of experimental means in verifying and improving computational gene prediction. For simplicity, we selected the gene prediction from the legume matrix for further analysis.

### Tiling microarray detection of predicted genes

**Figure 1:**
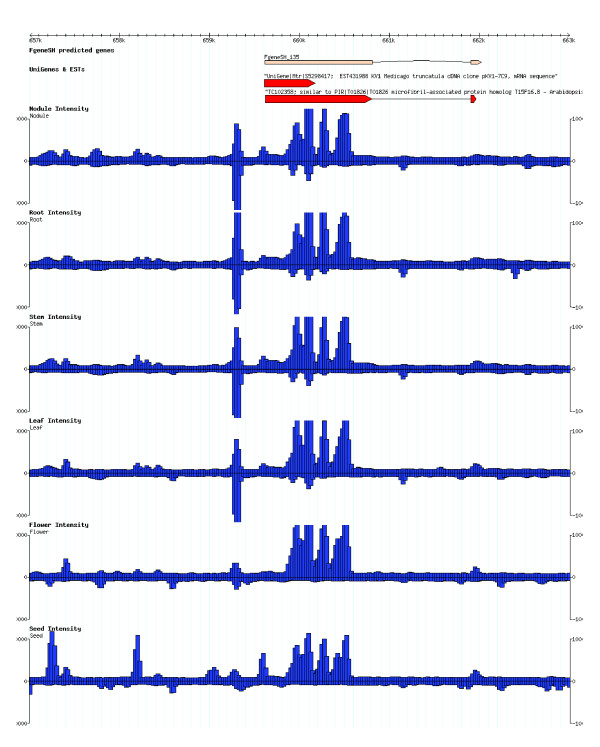
Tiling microarray analysis of the 1 Mb syntenic regions. A representative Gene Browser window is shown in which predicted genes are aligned to the chromosomal coordinates. Arrows indicate the direction of transcription. The interrogating tiling probes are also aligned to the chromosome coordinates with the fluorescence intensity value depicted as a vertical bar in the six organ types. From top to bottom: nodule, root, stem, leaf, flower and seed.

We designed two independent sets of overlapping 36-mer oligonucleotide probes offset by five nucleotides to represent both DNA strands of the 1 Mb syntenic barrel medic and soybean sequences (see Materials and methods). Each set of probes was synthesized into a single array based on Maskless Array Synthesis technology [8-10,34]. The barrel medic and soybean arrays were hybridized in parallel with target cDNA prepared from six organ types of each plant, namely, root, nodule, stem, leaf, flower and developing seed. Fluorescence intensity of the probes was correlated with the genome position by alignment of the probes to the chromosomal coordinates (Figure 1). Transcriptional analysis of the syntenic regions was then achieved by examining expression of the predicted genes and systematically screening for TARs.

**Figure 2:**
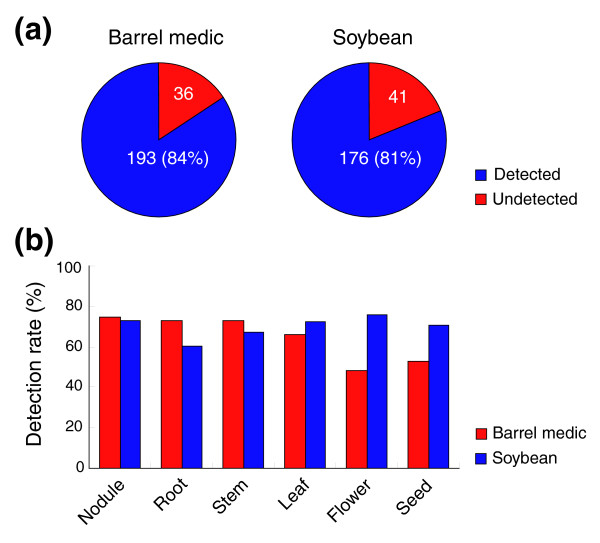
Tiling microarray detection of the predicted genes in the 1 Mb region syntenic between barrel medic and soybean. **(a) **Pie charts showing the number and percentage of genes detected by tiling arrays in at least one of the six examined organ types. **(b) **Tiling array detection rates of predicted genes in the six organ types in barrel medic and soybean.

We used a method based on the binomial theorem to score the tiling array data obtained from the six organ types to detect transcription of the predicted genes [10]. Analysis of the tiling array data detected 193 out of 229 (84%) and 176 out of 217 (81%) predicted genes in at least one of the six organ types in barrel medic and soybean, respectively (Figure 2a), indicating that most predicted gene loci are transcribed. Among the six organ types, detection rates of predicted genes ranged from 48% (flower) to 75% (nodule) in barrel medic, and from 60% (root) to 76% (flower) in soybean (Figure 2b). Interestingly, the gene detection rate in the nodule was the most similar between both species (74.7% and 73.3% in barrel medic and soybean, respectively; Figure 2b). These results suggest that transcription of the predicted genes from the 1 Mb syntenic sequences between barrel medic and soybean is, to a large extent, differentially regulated in the two species, which was further investigated (see below).

### Identification and characterization of TARs

**Figure 3:**
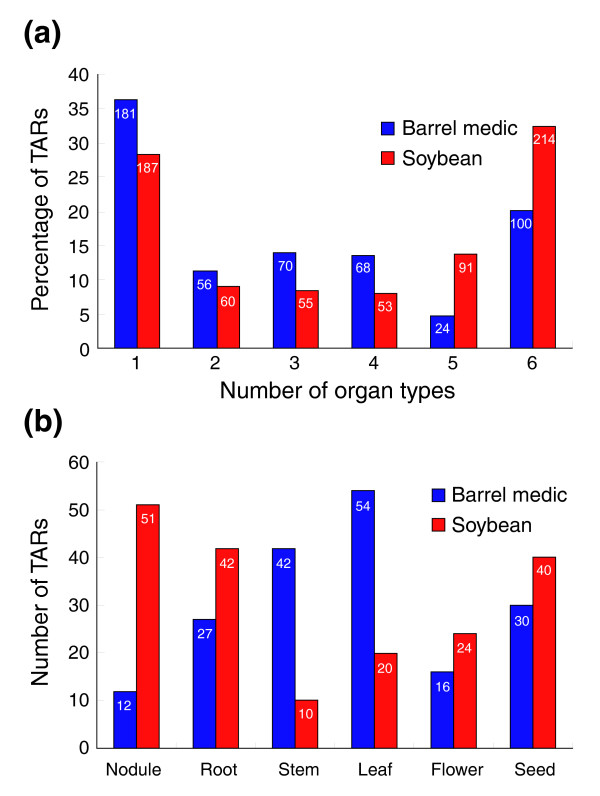
Analysis of the frequency of TARs in different organ types. **(a) **Percentage and number of TARs detected by tiling arrays in one, two, three, four, five and all six organ types in barrel medic and soybean. **(b) **Organ-specific number of TARs detected from only one organ type by tiling arrays in barrel medic and soybean.

We next scored tiling microarray data blind to the annotated genes and identified 499 and 660 unique TARs in barrel medic and soybean, respectively (see Materials and methods). The barrel medic and soybean TARs exhibited distinct overall organ specificity. Compared with TARs in barrel medic, soybean TARs in general were detected in more tissue types (Figure 3a), implying a more constitutive expression pattern. Furthermore, roughly equal numbers of barrel medic (181) and soybean (187) TARs were detected in just one organ type. These TARs were detected in barrel medic mainly from stem and leaf while nodule and root were the most abundance source in soybean (Figure 3b). Thus, these TARs appear to represent organ-specific transcriptional activities that differ in the examined sequences between barrel medic and soybean.

**Figure 4:**
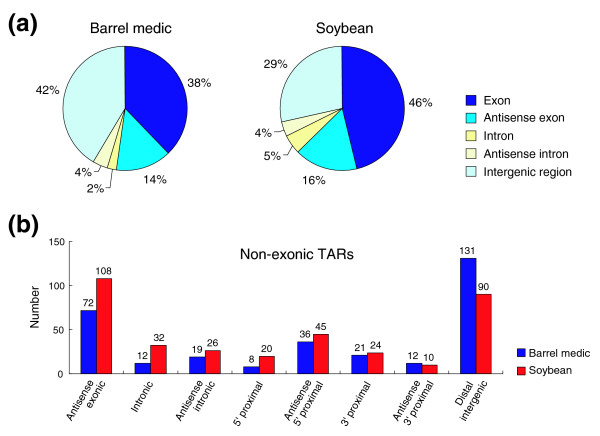
Classification of TARs based on physical location relative to the predicted genes. **(a) **Pie charts showing percentage of all identified TARs in different genome components relative to the predicted gene structures in barrel medic and soybean. **(b) **Number of non-exonic TARs in different sub-genic regions in barrel medic and soybean.

Aligning against the predicted genes, 188 (38%) and 305 (46%) barrel medic and soybean TARs intersect with an exon. The remaining 311 (62%) barrel medic and 355 (54%) soybean TARs are located outside of or antisense to the predicted exons and are referred to as non-exonic TARs. The distributions of TARs detected in barrel medic and soybean in the different annotated genome components are illustrated in Figure 4a. Interestingly, the relative proportion of TARs in each annotated genome component is largely comparable to results from a whole-genome tiling array analysis in rice [10]. This observation indicates that predicted exons account for less than half of the transcriptome detected by tiling arrays in rice and legume plants, despite their different genome sizes and distinct genome organization. Furthermore, a significant portion of TARs was found antisense to the predicted genes in both barrel medic (14%) and soybean (16%) (Figure 4a), which adds to previous tiling array analysis in *Arabidopsis *[5] and rice [8-10] in showing that antisense transcription is an inherent property of the plant genomes.

The non-exonic TARs were further analyzed in terms of their physical location relative to the predicted genes. In this analysis, genome regions were divided into eight different configurations against the predicted exons (Figure 4b). Interestingly, in almost all antisense configurations, there were more TARs in soybean than in barrel medic (Figure 4b), suggesting that antisense transcription is more prevalent in soybean than in barrel medic. This analysis also revealed a surprisingly large number of intergenic TARs (36 in barrel medic and 45 in soybean) located in close proximity on the antisense strand 5' to the start of a predicted gene (Figure 4b). Because the predicted genes do not include untranslated regions, it is conceivable that transcripts derived from these TARs and the corresponding genes are arranged in a divergent antisense orientation and could potentially form duplex transcript pairs.

### Differential gene expression in the syntenic regions

**Figure 5:**
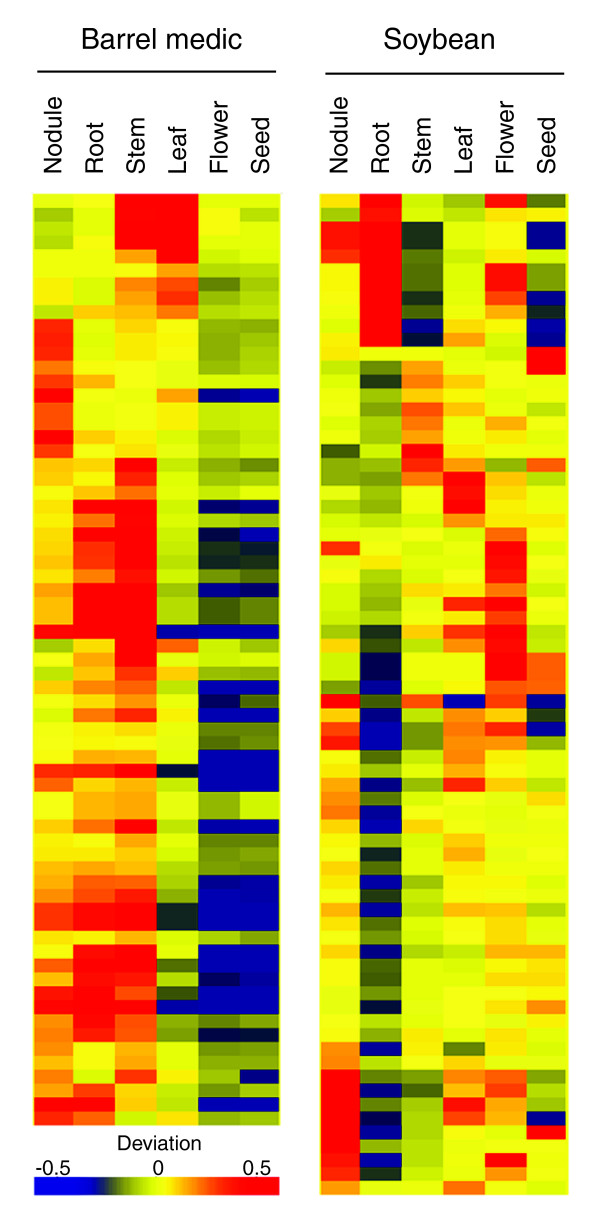
Analysis of differentially expressed genes. Heat maps represent unsupervised clustering of differentially expressed genes in barrel medic and soybean. The red, yellow, and blue colors depict positive deviation, no deviation, and negative deviation of the transcription level, respectively.

The binomial theorem-based method used to detect gene transcription does not assign a value to the expression level and is only useful for present calls [35]. Therefore, we used a median polishing-based method that fits an additive linear model [36] to determine differential expression of the predicted genes in the six examined organ types and to assess the relative deviation of gene expression level in each organ type (see Materials and methods). In barrel medic, 67 (29%) of the 229 predicted genes were identified as differentially expressed (*p *< 0.001) among the six examined organ types (Figure 5). In soybean, 72 (33%) of the 217 predicted genes displayed differential expression (Figure 5).

**Figure 6:**
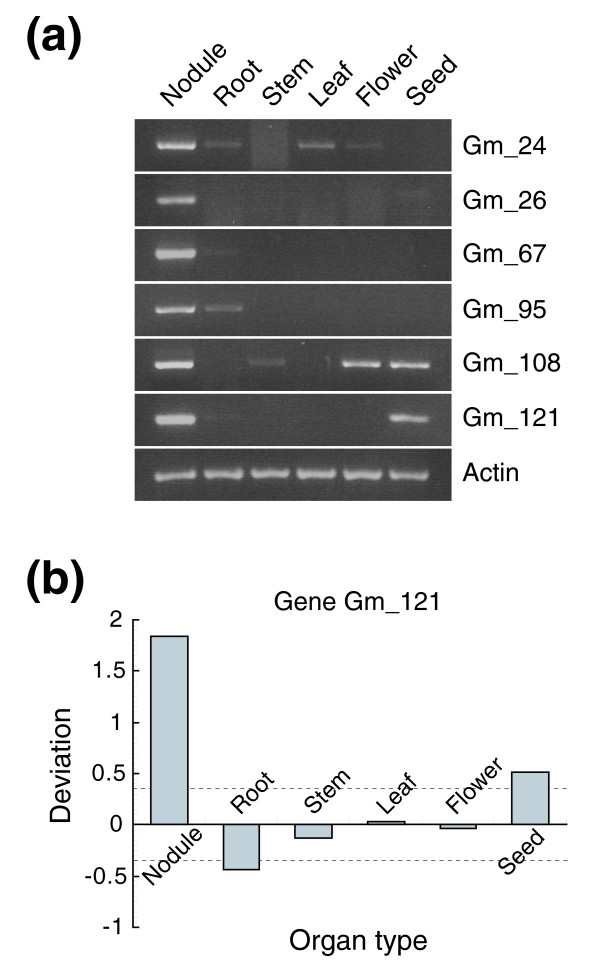
Verification of tiling array detected differentially expressed genes. **(a) **RT-PCR analysis of the transcript abundance in six organ types for six selected soybean genes that are preferentially expressed in the nodule. Total RNA (5 μg) was reverse transcribed and 5% of the product used as template for PCR, which was carried out for 35 cycles. **(b) **Organ type-specific variation of the expression level of the gene Gm_121, as determined by median-polishing of the tiling array data. Dashed lines indicate the deviation value at *p *= 0.001.

Precise transcriptional and developmental controls are required for the establishment of the complex interaction between the nitrogen-fixing rhizobia and plant cells in the nodule. To begin to understand the transcriptional program in nodules, we identified and compared genes specifically expressed in the nodule. Within the syntenic regions in barrel medic, 11 (16%, including one duplicated gene) differentially expressed genes showed higher transcription levels in the nodule than in the other five organ types (Additional data file 2). In soybean, there were 10 (14%) differentially expressed genes showing higher transcription levels in the nodule (Additional data file 3). Nodule-enhanced expression levels of six randomly selected genes in soybean were all confirmed by RT-PCR analysis (Figure 6a), indicating that the median polishing-based method used to score the tiling data is accurate in detecting organ type-specific transcripts. A particular example is illustrated in Figure 6b. This gene (Gm_121) is homologous to the *Ljsbp *gene from *Lotus japonicus *that encodes a putative selenium binding protein [37]. *In situ *hybridization analysis revealed that the *Ljsbp *transcripts were localized in the young nodules, the vascular tissues of young seedpods and embryos [37], which is consistent with the tiling array and RT-PCR data on the soybean ortholog (Figure 6b).

In soybean, all but one of the detected nodule-enhanced genes are known genes (Additional data file 3). In contrast, only three of the 11 nodule-enhanced genes detected in barrel medic match with a known gene while the other eight genes have no assigned functions (Additional data file 2). When the nodule-enhanced genes detected in barrel medic and soybean were compared for synteny, six of the ten soybean genes were found to have a collinear counterpart in barrel medic, although transcription of the collinear genes in barrel medic was not nodule-enhanced (Additional data file 3). Consequently, there was only one gene encoding a TGACG-binding transcription factor that is collinear as well as specifically expressed in the nodule in both species.

### Transcriptional pattern of collinear genes in the syntenic regions

The barrel medic and soybean sequences interrogated by the tiling microarray are highly syntenic. In the previous report, a total of 68 pairs of genes were found to be collinear with both the gene order and orientation conserved between barrel medic and soybean homologs [32]. In the current study, we were able to identify 78 collinear gene pairs based on the gene prediction output from the legume matrix.

**Figure 7:**
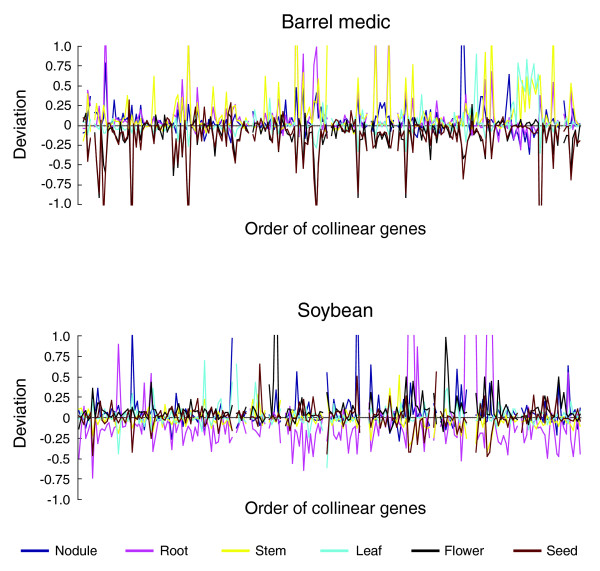
Analysis of the transcription patterns of collinear genes. The collinear genes in both barrel medic and soybean are ordered by chromosome position. For each gene, the deviation of transcription level was calculated based on median polishing for the six organ types (see Materials and methods). The gene order was then plotted against the corresponding deviation value in each of the six organ types, which is color-coded.

To begin to obtain information on the variation in gene expression between barrel medic and soybean, which is important for defining transcriptional regulatory networks that contribute to their phenotypic variations [38], we examined the expression pattern of the collinear genes. To this end, we used the transcription level deviation in the six organ types for each collinear gene as a parameter to profile gene expression patterns. Consistent with the fact that most genes were not differentially expressed in different organ types, a majority of the collinear genes showed relatively small organ type deviation (Figure 7). However, a number of collinear genes exhibited drastic variation in transcription levels across the organ types. In barrel medic, the most conspicuous example is a group of genes that are down-regulated in the seed but up-regulated in the stem. In soybean, the root exhibited the greatest gene expression variation (Figure 7). Importantly, the transcription pattern of these collinear genes is not conserved in the reciprocal species, suggesting that the regulatory sequence of these genes is under positive selection.

## Discussion

The rapidly accumulating amount of genome and transcriptome data in recent years is having profound effects on biological research. Elucidating all the functional and structural elements of the genome sequences and how they are organized and regulated, and how they evolved has thus become the focus of the next phase of genome projects. In these regards, genome tiling microarray analysis is emerging as a new powerful approach, which involves the development of tiling arrays containing progressive oligonucleotide tiles that represent a target genome. Recent advances in microarray technologies allow oligonucleotide arrays to be made with several hundred thousand to several million discrete features per array, which permits tiling complex genomes with a manageable number of arrays [1,2]. This in turn has resulted in transcriptomic tiling data for a large number of model species [1-10].

Application of tiling array analysis in genomics studies has significantly broadened our understanding of the genetic information encoded in the genome sequences. When probed against various RNA samples, tiling array hybridization patterns identify transcript ends and intron locations [3-8]. Tiling array analysis thus provides a valuable means for verifying genome annotation, which is a challenge that must be met for each new genome sequence. In the current study, we generated tiling array data for a 1 Mb region syntenic between barrel medic and soybean in six different organ types (Figure 1). Analysis of the tiling array data detected 193 out of 229 (84%) and 176 out of 217 (81%) predicted genes in barrel medic and soybean, respectively (Figure 2), similar to results reported from tiling array analysis of the rice genome [8,9]. Because genome annotation is a highly reiterative process that improves with the parallel refinement of gene-finding programs and the availability of experimental evidence, we anticipate further application of tiling array analysis to facilitate annotation of the fast emerging legume genome sequences [25,30,39].

Another use of the tiling array data is to identify transcription units in addition to the predicted genes [1,2]. Previous tiling analyses indeed documented large numbers of putative novel transcripts in virtually all the genomes examined [3-10]. For example, detailed characterization of the non-exonic TARs identified in the *japonica *rice genome showed that they could be assigned to various putatively functional or structural elements of the genome, ranging from splice variants, uncharacterized portions of incompletely annotated genes, antisense transcripts, duplicated gene fragments, to potential non-coding RNAs [10]. In carrying out tiling array analysis of the legume sequences, we identified 499 and 660 unique TARs in barrel medic and soybean, respectively (Figure 3). Aligning against the predicted genes, 311 (62%) barrel medic and 355 (54%) soybean TARs were found to locate outside of or antisense to the predicted exons (Figure 4). Interestingly, in a promoter trapping study in lotus in which a promoter-less GUS reporter system was used, GUS activation, often tissue-specific, was found beyond the predicted genic regions [40]. Together, these observations indicate that novel transcripts missed by gene annotation account for a significant portion of the transcriptome in legume plants.

As a novel use of tiling array data for transcriptomic profiling, we used a median polishing-based procedure [10,36] to determine the relative transcription levels and differential expression of the predicted genes. Because there are multiple probes involved in tiling a given gene, the median polishing-based procedure will have the corollary benefit of improved statistical confidence. Based on this method, approximately 30% of genes were found to be differentially expressed among the six examined organ types (Figure 5). The nodule-enhanced expression pattern of six selected soybean genes was subsequently verified by RT-PCR analysis (Figure 6). Collectively, these results indicate that genomic tiling array analysis can be extended to quantitatively examine the transcription levels of individual genes. This may prove particularly useful for quantifying transcription levels of members of paralogous gene families, which are notoriously hard to discriminate in conventional expression arrays that employ relatively fewer probes per gene.

Interestingly, 11 and 10 genes were identified as preferentially expressed in the nodule in barrel medic and soybean, respectively. These genes exhibited little overlap between the two species (Additional data files 2 and 3). Barrel medic and soybean diverged from a common ancestor approximately 50 million years ago, and represent two distinct groups of nodulating plants [41]. Barrel medic forms indeterminate nodules, which maintain an active meristem inside nodule primordia during the early stages of nodule development; while soybean forms determinate nodules that, after initial cell divisions, grow by cell expansions. These morphological differences may thus affect the architecture and gene expression in the nodules [42].

The availability of multiple genomic and transcriptomic datasets fosters comparative analyses that improve structural annotation and generate new insight into the function and evolution of coding and non-coding regions of the genomes [43]. A major principle of comparative genomics is that the functional DNA sequences in related species conserved from the last common ancestor are preserved in contemporary genome sequences, which encode the proteins and RNAs and the regulatory sequences controling genes with similar expression patterns [43]. Alignment of primary DNA sequences is the core process in most comparative analyses. The resulting information on sequence similarity among genomes is a major resource for infering gene functions, identifying other candidate funcational elements, and finding conserved genes missed from annotation in one genome or another.

Transcriptomic data from multiple species are also being extensively used in comparative analysis. For example, direct comparison of multiple transcript datasets using genome annotation tools has been shown as an effective way to uncover 'unannotated' genes. In rice, 255 new candidate genes were identified by cross-species spliced alignment of expressed sequence tags and cDNA to the genome sequence [44]. In this regard, the rich transcriptional activity documented from genomic tiling analysis constitutes an excellent complement to other tag-based transcriptome data. In the present tiling analysis of syntenic regions between two legume species, we identified over 300 unique TARs in both barrel medic and soybean in addition to the predicted exons. Transcripts tagged by these TARs should be useful for further comparison aimed at improving genome annotation and elucidating the transcriptome.

Furthermore, comparison of transcription levels in six different organ types revealed that a large portion of the collinear genes between barrel medic and soybean exhibit different expression patterns (Figure 7). It should be noted that there is a segmental duplication of synteny block 2 (block 2i and 2ii) in barrel medic [32]. The process of subfunctionalization following gene duplication, where degenerative mutations in both genes result in the partitioning of ancestral functions or expression patterns in the duplicated genes, could, therefore, contribute to the observed expression divergence among the examined collinear genes between barrel medic and soybean. Further analysis of the *cis*-regulatory regions of the syntenic genes should help to identify the key regulatory sequence divergence that accounts for the differences in related legume species and add to our general knowledge of plant genome evolution and regulation.

## Conclusion

We report here a transcriptional analysis using high-resolution tiling microarrays of syntenic regions totaling 1 Mb between the legume plants barrel medic and soybean in six different organ types. This analysis generated transcriptomic data that is useful for three purposes. First, we detected transcription of over 80% of the predicted genes in the interrogated genome regions in both legume species. As genome annotation is a reiterative process that is heavily dependent on experimental data, genomic tiling analysis is thus one valid option to meet the challenge of analyzing large-scale transcriptomic datasets for newly sequenced legume genomes. Second, we identified 499 and 660 TARs from barrel medic and soybean, respectively, over half of which are outside of the predicted exons. Further functional characterization of these candidate transcripts should be useful to better our understanding of the complexity and dynamics of the transcriptome of legume plants. Third, we used the tiling array data to detect differential gene expression and to compare transcription patterns of collinear genes. This novel approach was validated by the high confirmation rate by RT-PCR analysis of genes that are preferentially expressed in the nodule. Further investigation revealed that some collinear genes exhibited drastically different transcription patterns between the two species. Collectively, these results demonstrate that genomic tiling analysis is an effective approach to simultaneously complement computational annotation of newly available genome sequences and to facilitate comparative genomics aimed at elucidating genome organization and transcriptional regulation in closely related species.

## Materials and methods

### Plant materials and treatments

Barrel medic (*Medicago truncatula *cv. *Jemalong A17*) seed was treated with concentrated H_2_SO_4 _for 10 minutess, rinsed with water and then allowed to germinate on moist filter paper at room temperature for a week. Seedlings 1-2 cm in length were planted in soil and maintained in the greenhouse with nitrogen-free plant nutrient solution as previously described [45]. Soybean (*Glycine max *cv. *William 82*) seed was directly sown in soil and maintained in the greenhouse with nitrogen-free plant nutrient solution as described by Subramanian *et al*. [46].

The *Rhizobium *bacterium *Sinorhizobium meliloti *1021 and *Bradyrhizobium japonicum *USDA110 was used to inoculate barrel medic and soybean plants, respectively. The bacteria were grown in a yeast extract-mannitol medium for three days at 28°C as previously described [47]. The bacterial cells were then suspended in nitrogen-free nutrient solution to an OD_600 _of 0.08 and used to water four-week-old plants. This flood-inoculation step was repeated after two weeks. The nodules were collected three weeks after the second treatment. Each nodule was separated from the roots with sharp tweezers and placed on dry ice immediately. The stem, root, and leaf organs were harvested from four-week-old plants that were maintained with nitrogen containing plant nutrient solution. The same plants were maintained until maturity for collection of the flower and seed organs.

### Sequence selection and gene prediction

Soybean sequences from four bacterial artificial chromosomes (BACs) were obtained from GenBank (accession numbers: AX196294.1, AX196295.1, AX196297.1, and AX197417.1). The BACs AX196294 and AX196295, and AX196297 and AX197417 form two contigs. There is a physical gap (represented by 100 Ns) between AX196294 and AX196295, and an approximately 50 Kb overlap between AX196297 and AX197417. Thus, the two contigs represent a total of 977 Kb of non-redundant sequences. Putative homologs to these soybean sequences in barrel medic were identified from sequenced barrel medic BACs as previously reported [32]. A total of 12 BACs (accession numbers: AC141115.22, AC149303.10, CR378662.1, CR378661.1, AC142498.20, AC146585.18, AY224188.1, AC146706.8, AY224189.1, AC146705.11, AC144644.3, and AC146683.9) were identified. The sequences were aligned and merged in regions of sequence overlap on the basis of ≥99% identity [32]. The merged barrel medic sequences form 7 contigs and total 1,060 Kb. Genes were predicted from soybean and barrel medic sequence contigs using FGENESH [33]. Both the dicot plants (*Arabidopsis*) and the *Medicago *(legume plant) matrix was used and their output compared.

### Tiling microarray design, production, and hybridization

Both the barrel medic and soybean tiling arrays were produced on the Maskless Array Synthesizer platform as previously described [6,8,34]. Briefly, tiling paths consisting of 36-mer oligonucleotides offset by five nucleotides were designed to represent both DNA strands of the selected barrel medic and soybean genome sequence. Probes were synthesized at a feature-density of 390,000 probes per array in a 'chessboard' design [34]. Microarray production and storage were carried out previously described [6,8,34].

Total RNA and mRNA were sequentially isolated using the RNeasy Plant Mini kit (Qiagen, Valencia, CA, USA) and the Oligotex mRNA kit (Qiagen), respectively, according to the manufacturers' recommendations. mRNA from different organ types was reverse transcribed using a mixture of oligo(dT)_18 _and random nonamer primers [6,34], during which amino-allyl-modified dUTP (aa-dUTP) was incorporated. The aa-dUTP decorated cDNA was fluorescent labeled by conjugating the monofunctional Cy3 dye (GE Healthcare, Piscataway, NJ, USA) to the amino-allyl functional groups in the cDNA. We used 2 μg dye-labeled targets for hybridization as previously described [6,8,34]. Tiling microarray design and experimental data are available in the NCBI Gene Expression Omnibus under the SuperSeries GSE10151, which is composed of subset series GSE10055 and GSE10056 for the barrel medic and soybean arrays, respectively.

### **Tiling array analysis of gene expression**

Raw microarray data were first Log_2 _transformed and then quantile normalized for the six organ types. A sign-test was used to determine transcription of the predicted gene [10,35]. First, probes lying within the exons of a predicted gene were checked to determine if their intensity was greater than the median of all probes in the array. Next, we determined whether or not the number of probes with intensity above the median was more than expected by chance alone. The probability, p, of obtaining h probes with intensity above median out of N probes is given by the equation:

$$\text{p}=\text{0}{\text{.5}}^{\text{N}}\;\sum_{\text{i=h}}^{\text{N}}\left(\begin{array}{c} \text{N}\\ \text{i} \end{array}\right)$$

Finally, genes with a *p*-value smaller than 0.05 were considered as detected.

Determination of differential gene expression was carried out based on the Tukey's median polish procedure, which fits an additive linear model [10,36]. The expression level of a given gene in the six organ types, *M*, is estimated by the equation:

*O*_*ij *_= *M *+ *a*_*i *_+ *v*_*j *_+ *e*_*j*_

where *O*_*ij *_is the observed intensity of the *i*^th ^probe in the *j*^th ^organ type, *a*_*i *_is the probe affinity effect of the *i*^th ^probe, *v*_*j *_is the organ type variation of the *j*^th ^organ type, and *e*_*j *_is the experimental error. A series of iterations of residue subtraction was performed using Tukey's median polishing until the matrix reaches a stable status, and the organ type deviation *v *was determined for each organ type. To obtain the false negative error rate, a permutation test was applied. The intensities of every probe were randomly shuffled among the organ types and median polishing repeated 1,000 times. A significant level at *p *< 0.001 was selected for organ-specific expression calling.

### Identification of TARs

To identify TARs in each organ type, the Log_2_-transformed, quantile-normalized data that were anchored to the chromosome coordinates were scanned from the 5' end of each DNA strand one probe at a time. TARs were identified as regions with length >70 nucleotides (spanning roughly 14 probes) with no 2 consecutive probes having an intensity below the cutoff of 11.0.

### Identification of collinear genes

To identify collinear genes, repetitive sequences were masked and tandemly duplicated genes were counted as one. The BLAT [48] program was then used to search the protein sequences of the predicted genes between barrel medic and soybean. The gene pairs with more than 10 amino acids mapped and more than 40% identity over the entire mapped sequences were identified as potential collinear genes, which were further manually inspected for gene order and orientation.

## Abbreviations

BAC, bacterial artificial chromosome; TAR, transcriptionally active region.

## Authors' contributions

X-WD and NDY conceived the project; X-WD and LL designed the research; LL performed experiments; HH, LL, XW, VS, and WT analyzed the data; LL wrote the paper; JZ, OY, and SB provided plant materials.

## Additional data files

The following additional data are available. Additional data file 1 is a table showing a summary of predicted genes in the 1 Mb syntenic regions between *Medicago truncatula *and *Glycine max*. Additional data file 2 is a table listing the *Medicago truncatula *genes preferentially expressed in the nodule. Additional data file 3 is a table listing *Glycine max *genes preferentially expressed in the nodule.

## Supplementary Material

Additional data file 1 Predicted genes in the 1 Mb syntenic regions between *Medicago truncatula *and *Glycine max*. Click here for file

Additional data file 2 *Medicago truncatula *genes preferentially expressed in the nodule. Click here for file

Additional data file 3 *Glycine max *genes preferentially expressed in the nodule. Click here for file
