# Supplementary material for: Transcriptional analysis of highly syntenic regions between Medicago truncatula and Glycine max using tiling microarrays
Source: Genome Biol. 2008 Mar 19;9(3):R57. doi: 10.1186/gb-2008-9-3-r57 (PMC2397509; doi:10.1186/gb-2008-9-3-r57)
Supplement: Additional data file 1 — Predicted genes in the 1 Mb syntenic regions between Medicago truncatula and Glycine max. [file gb-2008-9-3-r57-S1.pdf]

Table S1. Summary of predicted genes in the syntenic regions between *Medicago truncatula* and *Glycine max*.

Species	Matrix [a]	Gene Number	Average Gene Length [b]	Exon Number	Average Exon Length [b]	Average Intron Length [b]	Total Exon Length [b]
Barrel medic	Legume	229	897	829	248	396	205,592
	Dicot	153	1216	714	261	359	186,354
Soybean	Legume	217	975	818	259	417	211,862
	Dicot	156	1254	702	279	386	195,858

[a] Organism selected when use FGENESH.

[b] In bases.
