# Supplementary material for: Transcriptional analysis of highly syntenic regions between Medicago truncatula and Glycine max using tiling microarrays
Source: Genome Biol. 2008 Mar 19;9(3):R57. doi: 10.1186/gb-2008-9-3-r57 (PMC2397509; doi:10.1186/gb-2008-9-3-r57)
Supplement: Additional data file 2 — Medicago truncatula genes preferentially expressed in the nodule. [file gb-2008-9-3-r57-S2.pdf]

Table S2. *Medicago truncatula* genes preferentially expressed in the nodule.

Gene	Strand	*v*_Nodule	*v*_Root	*v*_Stem	*v*_Leaf	*v*_Flower	*v*_Seed	*p* = 0.001	Collinear	Unigene Match
Mt_6	-	0.36	0.25	-0.07	0.07	-0.18	-0.16	0.23	Gm_24	EST649123 KV3 and cDNA clone KV3-53I24
Mt_17	-	0.32	0.02	0.07	-0.02	-0.11	-0.07	0.14	/	cDNA clone MHRP-17G5
Mt_95	+	0.32	0.13	0.02	-0.02	-0.05	-0.06	0.29	/	No
Mt_138	-	0.28	-0.01	0.01	0.05	-0.09	-0.08	0.15	/	No
Mt_139	-	0.22	0.03	0.02	-0.02	-0.17	-0.13	0.2	/	No
Mt_140	-	0.28	-0.01	0.01	0.05	-0.09	-0.08	0.15	/	No
Mt_176	-	2.95	0.01	-0.01	0.16	-0.42	-0.68	2.21	/	No
Mt_184	+	0.36	-0.03	0.06	0.03	-0.19	-0.14	0.18	/	No
Mt_185	+	0.36	-0.03	0.09	0.03	-0.18	-0.19	0.15	/	EST397679 KV0 and cDNA clone pKV0-20E5
Mt_196	-	0.38	-0.04	0.06	0.04	-0.19	-0.16	0.18	/	No
Mt_197	+	0.64	0.1	0.05	-0.05	-0.13	-0.09	0.28	/	No
