# Supplementary material for: Transcriptional analysis of highly syntenic regions between Medicago truncatula and Glycine max using tiling microarrays
Source: Genome Biol. 2008 Mar 19;9(3):R57. doi: 10.1186/gb-2008-9-3-r57 (PMC2397509; doi:10.1186/gb-2008-9-3-r57)
Supplement: Additional data file 3 — Glycine max genes preferentially expressed in the nodule. [file gb-2008-9-3-r57-S3.pdf]

Table S3. *Glycine max* genes preferentially expressed in the nodule.

Gene	Strand	*v* _Nodule	*v* _Root	*v* _Stem	*v* _Leaf	*v* _Flower	*v* _Seed	*p* = 0.001	Collinear	Unigene Match
Gm_9	-	0.36	-0.32	-0.08	-0.01	0.18	0.01	0.24	/	cDNA clone Gm-c1027-604, HYPOTHETICAL OXIDOREDUCTASE
Gm_24	+	1.06	-0.37	-0.13	0.3	0.13	-0.42	0.33	Mt_6	TGACG-motif binding protein (STGA1)
Gm_26	-	0.2	-0.11	0.02	0.09	-0.02	-0.02	0.15	/	cDNA clone Gm-r1083-1994
Gm_67	+	0.97	-0.24	-0.15	0.43	0.15	-0.16	0.19	Mt_88	cDNA clone Gm-r1083-3116
Gm_95	-	0.56	-0.18	-0.11	-0.01	0.01	0.01	0.24	/	cDNA clone Gm-c1036-11538, AMIDASE-LIKE PROTEIN
Gm_108	+	0.55	-0.28	0.28	-0.61	0.31	-0.44	0.39	/	Brassinosteroid-regulated protein mRNA
Gm_118	-	0.19	-0.25	-0.05	0.02	-0.02	0.08	0.16	Mt_179	Aspartokinase-homoserine dehydrogenase (AK-HSDH)
Gm_121	+	1.84	-0.44	-0.13	0.03	-0.03	0.51	0.35	Mt_137	Partial mRNA for selenium binding protein
Gm_127	-	0.64	-0.41	-0.27	0.12	0.31	-0.12	0.46	Mt_135	cDNA clone Gm-c1049-6968, PUTATIVE MICROFIBRIL-ASSOCIATED PROTEIN
Gm_164	-	0.59	-0.24	-0.21	0.2	0.26	-0.2	0.46	Mt_158	No
